# Effects of *Ottonia anisum* plant extract on local anesthetic, analgesic, anti-inflammatory and HCl‑induced acute lung injury activities: a study in animal models

**DOI:** 10.1186/s40643-023-00706-8

**Published:** 2023-11-30

**Authors:** Mingming Liu, Hui Wang, Qiang Yue, Junli Liu

**Affiliations:** Department of Anaesthesiology, Xiangyang Central Hospital, Affiliated Hospital of Hubei University of Arts and Science, No. 136 Jingzhou Street, Xiangcheng District, Xiangyang City, 441021 Hubei Province China

**Keywords:** *Ottonia anisum*, Local anesthetic agent, Acute lung injury, Proinflammatory molecules, Redox homeostasis, Analgesic

## Abstract

*Ottonia anisum *(*O. anisum*), belonging to the family Piperaceae, is renowned for its medicinal properties. The plant is rich in alkaloids, terpenoids and flavonoids with recorded bioactivities. The stems, roots, and leaves, of the *O. anisum* have been extensively used in the folk medicine. Therefore, the present study was conducted to examine the pharmacological activities of *O. anisum* root extract. Methanolic root extract of *O. anisum* was assessed for local anesthetic, analgesic, anti-inflammatory and HCl-induced acute lung injury activities in animal models. Local anesthetic activity assessed in frog and guinea pigs through foot withdrawal reflex and intradermal wheal method, respectively, revealed the dose-dependent onset time of anesthesia response. In the case of HCl-induced ALI, the mice group orally administered with *O. anisum* extract were assessed for bronchoalveolar lavage fluid (BLF) contents, oxidative stress, and proinflammatory molecules. The analysis revealed the reduction in inflammatory molecules, neutrophils, and oxidative stress in the extract treated mice group. In addition, the redox homeostasis, reduced GSH and the catalase activity was found to be restored in the treated groups. Intriguingly, the genes associated with the NFkB expression was found to be downregulated in *O. anisum* extract treated groups. Moreover, the extract unveiled the significant analgesic and anti-inflammatory activities. Overall, the findings emphasize the clinical applicability of *O. anisum* extract in the treatment of ALI as well as the potential usage in local anesthetic, analgesic, and anti-inflammatory agents during the treatments.

**Graphical Abstract:**

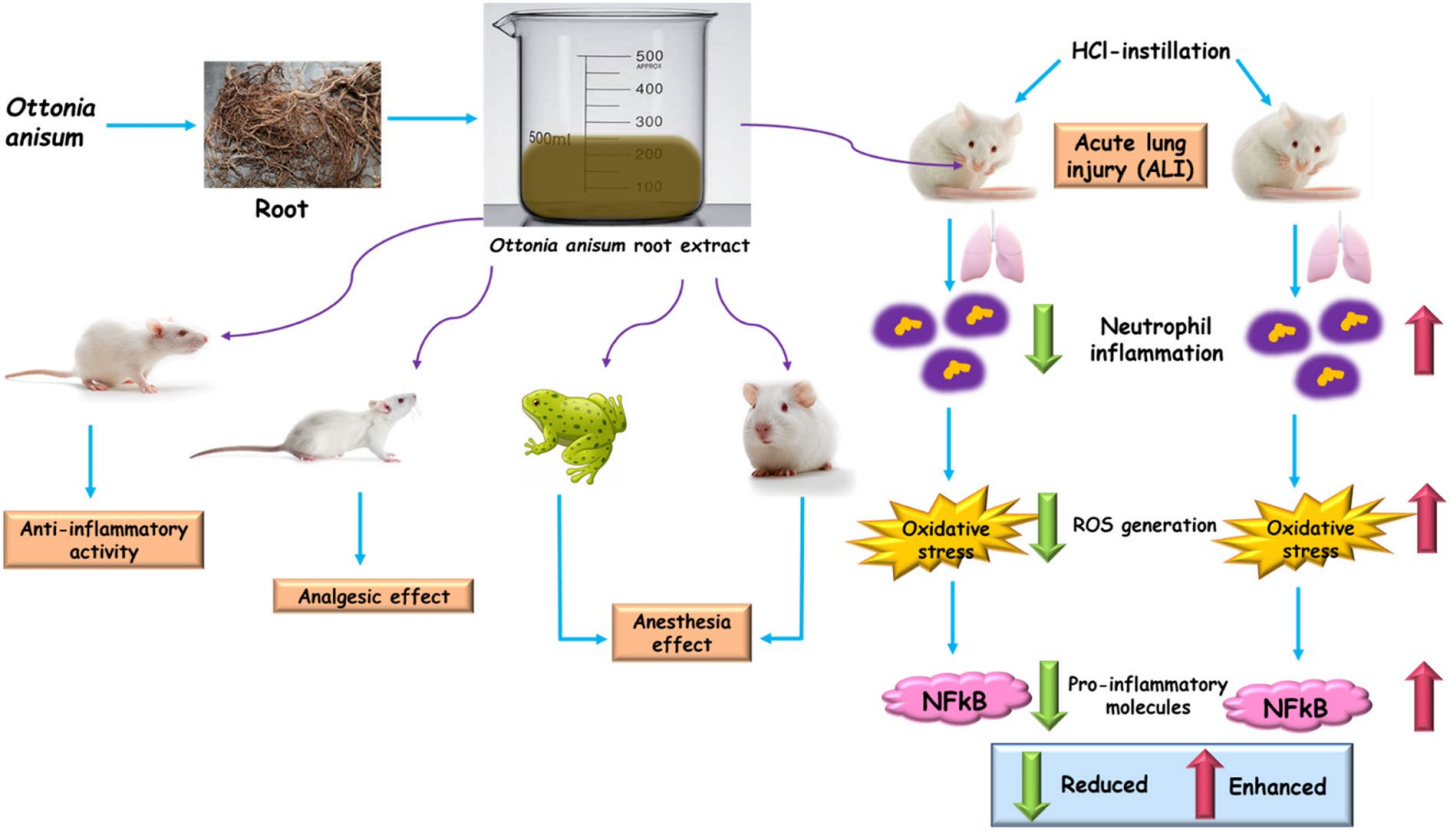

## Introduction

Due to constant exposure of lung to pollutants, allergens, irritants, and pathogens, the inflammatory response plays a vital role in defending against any invading foreign materials (Lloyd and Marsland 2017). However, lung homeostasis is highly essential to maintain the steadiness between inflammatory and anti-inflammatory state, because an inflammatory response due to increased vascular permeability have a decisive role in developing acute lung injury (ALI). ALI is associated with endothelial injury, accumulation of inflammatory cell, fluid leakage into alveolar spaces, and pulmonary interstitial edema. This severe inflammatory syndrome give rise to emphysema and chronic pulmonary disease, that account for 30–50% mortality. Nuclear factor-kappa B (NFkB), a DNA binding protein, plays a significant role in maximal transcription of proinflammatory molecules, including TNF-α, IL-6, and IL-1β (Tsushima et al. 2009; Butt et al. 2016; Johnson and Matthay 2010). Activation of NFkB has been shown to associate with the pathogenesis of inflammation, and lung cancer (Tang et al. 2006; Liu et al. 2017). Thus, inhibition of NFkB is thought to successfully block the inflammatory responses in ALI treatment. Intratracheal instillation of lipopolysaccharide (LPS) has been extensively used to investigate the proinflammatory cytokines (Wan et al. 2018; Zhu et al. 2013). Consequently, resulting in the massive creation of reactive oxygen species (ROS) in the lungs. Oxidative stress occurs due to imbalance between generation and accumulation of ROS, and particularly dysregulation in the antioxidant defense mechanisms (Kellner et al. 2017). Therefore, it is important to scavenge the generated ROS molecules to ameliorate the antioxidant capacity in the system.

Anesthetic agents are class of molecules that causes partial or complete loss of sensation. Anesthesia is defined as the state of loss of sensation and it can be classified into two types viz., general and local anesthesia. General anesthesia is the loss of sensation with depression in central nervous system, while local anesthesia is the shortfall of sensation without losing consciousness. Most of the anesthetic agents used today are derived from plant sources. Phytoconstituents such as alkaloids, flavonoids and terpenoids are always the choice of interest for anesthesia due to their mechanistical activities. These molecules are reported to network with ion-channels, lipid membranes and receptors to impede the sensory nerves or to hinder the movement of nerve impulses at the site of surgery. Cocaine, nerolidol, cineol, aconitine, epigallocatechin, morphine, and d-tubocurarine are some of the anesthetic agents derived from plant source (Tsuchiya 2017). Thus, medicinal plants are not only a rich source for phytoconstituents but also a rich source for anesthetic agents. Inflammation and pain are characteristic features of several diseases. It causes mild distress to severe death. Although corticosteroids, opioids, and other drugs are used to treat inflammation and pain, they cause severe side-effects. Thus, herbal medicinal plants have been explored for the therapeutic agents, including analgesic and anti-inflammatory activity (Olela et al. 2020). Indeed, plants are repertoire of drug molecules and therapeutically valuable products.

Piperaceae, a family of flowering plant contain more than 3000 species, grows in tropical and subtropical provinces of the biosphere. Plants belonging to this family are rich in phytoconstituents, such as terpenoids, flavonoids, alkaloids, glycosides, saponins, and steroids. The family piperaceae has been documented for innumerable biological activeness, such as anti-inflammatory, anti-angiogenic, antipyretic, antidiabetic, analgesic, anticancer, hypotensive, antioxidant, antimicrobial, larvicidal and immunostimulatory activities. Besides, it has been considered to treat illnesses, such as heart disease, hernia, joint pain, oral abscesses, lung and liver problems, diarrhea, indigestion etc. The plants from this family can be used for developing novel drug molecule, such as local anesthetic agents with promising biopharmaceutical activities (López et al. 2016; Cunico et al. 2015; Kuete et al. 2013; Raghavendra and Kekuda 2018; Marques et al. 2017). Therefore, the present study assesses the effect of *O. anisum* in the pharmacological activities of local anesthesia, analgesics, anti-inflammatory and HCl-induced ALI in experimental models.

## Materials and methods

### Chemicals and reagents

All the materials used in the current study were procured from Sigma-Aldrich, Shanghai.

### Animals

Male albino mice used in this research work were obtained and separated into four different groups and each group consisted of six animals. Animals were maintained at controlled temperatures and fed every day with food. Maintenance and handling of animals were carried out according to the guiding principles of the University Ethics Committee. The Animal ethics committee approved all the experiments.

### Preparation of crude extract from root bark

The root bark of *O. anisum* was collected from roadside, located nearby surroundings of Xiangyang Central Hospital, Xiangyang City, Hubei Province, China. The obtained root bark was washed (with sterile distilled water), dried and powdered. Around 10 g of powdered root bark was dissolved in 100 mL methanol and the mixture was continuously agitated at 30 °C for 16 h. After 16 h, the concoction was filtered using gauze and Whatman No1 filter paper (pore size of 150 mm in diameter) and the filtrate was collected in a flask. The remaining residues were treated repeatedly with fresh methanol until the extract becomes colourless. The collected filtrate was pooled and evaporated at room temperature (Batista et al. 2020). After, evaporation, the resulting dried flakes were stored and labelled as stock. Working solutions were prepared freshly during each experiment from the stock.

### Local anesthetic activity in frog

#### Foot withdrawal reflex

The experiments were performed at different concentrations of methanolic root bark extract of *O. anisum* (10, 20, 40, and 80 mg/mL) and five animals were used for each concentration. Frogs were decerebrated through pithing according to the protocol described previously (Ramanathan et al. 2011). In brief, the upper spinal cord, behind the eyes was destroyed. Then, the drug solution was filled in a sac created by dissecting the lateral region across the abdomen. Animals were placed on a board with head raised on forelimbs and hindlimbs relaxed. The reflex activity was monitored by placing one foot in 0.05 N HCl solution. The highest concentration of HCl solutions (0.1 and 0.2 N) were tried when there is no significant response from 0.05 N HCl solution (Ramanathan et al. 2011).

#### Intradermal wheal method in guinea pigs

Guinea pigs’ hairs were shaved and pricked with a pin on both sides to test the response of the animal upon treatment with 0.2 mL of methanolic root bark extract of *O. anisum*. A series of concentrations such as 10, 20, 40, and 80 mg/mL was used for evaluation. Guinea pigs were intradermally injected with methanolic root bark extract of *O. anisum* on the left side and normal saline (0.2 mL) on the right side and the injected sites were distinguished by ink. The animals were pricked six times at an interval of 2–3 s and after every 5 min pin pricks were repeated (Rates et al. 1997). Animals not responding to pin pricks were considered to be positive for anesthetic effect.

### Effect of O. anisum root extract in HCl-induced ALI mice

The *O. anisum *extract was suspended in 5% Tween 80 (prepared in 0.5% w/w carboxymethyl cellulose) and about 200 µL of the extract was used for oral administration (per mouse). Normal and healthy mice were used in this experiment. Mice were separated into 3 random groups. Group I mice was control and thus supplied with normal feed. Group II mice were subjected to intratracheal instillation with 0.1 N HCl (60 µL per mouse). For anesthetic purpose, xylazine/ketamine (10 mg per kg/100 mg per kg) were injected intraperitoneally. Group III mice were subjected to intratracheal instillation with 0.1 N HCl and extract. Different doses of extracts (50–200 mg/kg) were orally administered 90 min before HCl treatment. The doses were chosen based on the previous reports (Galani and Patel 2011; Patel et al. 2011). After 24 h time, the mice were sacrificed using diethyl ether and the lung tissues were collected. The bronchoalveolar lavage fluid (BALF) were also collected and for quantification of total protein content as well as total and differential leukocyte count in lung airways (Kapoor et al. 2015). Lung tissue homogenate was prepared for biochemical assays and simultaneously RNA was extracted from lung tissue for proinflammatory molecular analysis.

#### Analysis of biochemical parameters

Total protein estimation was carried out according to Lowry’s method (Lowry et al. 1951). A probe, 2′,7′-dichlorofluorescein diacetate (DCFD), was utilized to quantify ROS levels as described by Wang et al. (1999). The malondialdehyde (MDA), an important biomarker for oxidative stress, was assayed though the method defined by Ohkawa et al. (1979). Total glutathione (GSH) and catalase activity was assayed through Ellman’s (Ellman 1959; Zahler and Cleland 1968) and Aebi’s method (1984), respectively.

#### RNA extraction and RT-PCR analysis

The lung tissue was homogenized and the total RNA was extracted using TRIzol reagent as described by the manufacturer’s instructions. The extracted RNA was then converted into cDNA through a process known as reverse transcription using iScript™ cDNA Synthesis Kit (Bio-Rad). The synthesized cDNA was analysed for the proinflammatory response molecules expressions (TNF-α, IL-1 β, and ICAM-1) (Kapoor et al. 2015). To normalise the gene expression, β-actin was used as a reference gene. The primer details are provided in Table 1.

**Table 1:** List of primer sequence

Gene name	Forward primers	Reverse primers
IL-1β	5′-GACCTTCCAGGATGAGGACA-3′	5′-AGGCCACAGGTATTTTGTCG-3′
TNF-α	5′-TATGGCTCAGGGTCCAACTC-3′	5′-CTCCCTTTGCAGAACTCAGG-3′
ICAM-1	5′-AGCACCTCCCCACCTACTTT-3′	5′-AGCTTGCACGACCCTTCTAA-3′
β-actin	5′-TACAGCTTCACCACCACAGC-3′	5′-TCTCCAGGGAGGAAGAGGAT-3′

### Evaluation of analgesic activity

The protocol described by Woolfe and MacDonald (1944) was followed. In brief, Wistar albino rats were split into 5 groups with each group containing 6 rats. Group-I rats are vehicle control (supplied with Tween 80), while group-II rats are positive control (supplied with pentazocine). Group-III, IV and V are rats treated with *O. anisum* extracts of 50 mg/kg, 100 mg/kg and 200 mg/kg, respectively. The animals were injected intraperitoneally 30 min before positioning them on hot plate. In general, the rat paws are sensitive to heat. Therefore, upon exposure to heat it produces responses, such as jumping, licking or withdrawal of paws. The animals were habituated in advance to heat responses experiments. The rats were positioned on hot plate (55 ± 0.2 °C) with a cutoff time of 15 s. Latency was monitored at 30 min, 1 h, 1½ h and 2 h after the intraperitoneal injection. The following equation was used to calculate the percentage of analgesia:

$${\text{Percentage of analgesia}}=\frac{{{\text{TL}} - {\text{CL}}}}{{{\text{CL}}}} \times 100,$$

where TL is the latency of *O. anisum* extract treated rats, while CL is the latency of untreated rats.

### Evaluation of anti-inflammatory activity

The anti-inflammatory potential of *O. anisum* extract was evaluated through carrageenan paw oedema method (Chatterjee et al. 2015). In brief, the Wister rats were grouped into 5 with each containing 6 rats. Animals were orally administered with *O. anisum* extract before 1 h of sub-plantar carrageenan injection (1%, 100 μL in right hind paw). Inflammatory response elicited by carrageenan was analysed by measuring the paw volume at different time interval (from 0 to 3 h post-injection). Diclofenac served as positive anti-inflammatory agent. The following equation was used to calculate the percentage of anti-inflammatory activity:

$${\text{Percentage of anti-inflammatory activity}}= \frac{1-\mathrm{TR}}{\mathrm{CR}} \times 100,$$

where TR is the average paw volume of *O. anisum* treated groups and CR is the average paw volume of untreated groups.

## Results and discussion

### Local anesthetic activity in frog

#### Foot withdrawal reflex

**Fig. 1:**
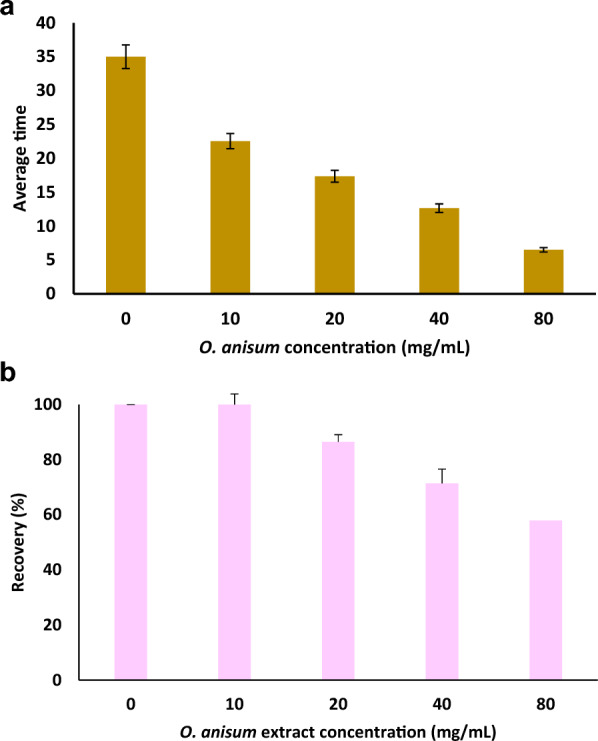
Evaluation of anesthetic effect of *O. anisum* extract in animal models. A concentration of 10 mg/mL to 80 mg/mL was examined. **A** Assessment through foot withdrawal reflex experiment in frog. **B** Evaluation of intradermal tracking in guinea pigs. Recovery rate is presented in percentages. Data are denoted as mean ± SD (*n* = 6)

To assess the anesthetic capacity of *O. anisum* root extract the foot withdrawal reflex experiments were performed in frog. Treatment with different concentrations of methanolic extract of root bark of *O. anisum* (10–80 mg/mL) revealed the dose-dependent onset time of anesthesia response (35.00, 22.54, 17.35, 12.64 and 6.49 min, respectively) in frog. As evident from one-way ANOVA (*P* value greater than 0.01, Fig. 1A), the local anesthetic activity was found to be highly significant. The onset time was observed to decrease with rising concentration of *O. anisum* extract. The results revealed the significant anesthesia activity at maximal concentrations in which the anesthesia effect lasted for about 25 min and within 30 min the effect reverted to normal state. These findings indicate the presence of pharmacologically active principle in *O. anisum* extract that could initiate the anesthesia effect in frog. Similar results were reported from the methanolic leaf extract of *Kalanchoe Pinnata* (Kumar et al. 2022) in where, the methanol extract of the *Kalanchoe Pinnata* was dose-dependently analgesic up to 83.79% against acetic acid-induced pain in mice. In another study, the methanolic leaf extract of *Bryophyllum pinnatum* was found to exhibit dose dependent anesthesia response (Kanti et al. 2020).

#### Intradermal tracking in guinea pigs

In the case of guinea pigs, treatment with 10 and 20 mg of *O. anisum* methanolic extract showed 21 and 17 min as average onset time of local anesthesia, while treatment with 40 and 80 mg/mL revealed 11 and 6.5 min as onset time of local anesthesia. Considerable response was observed at guinea pigs treated with 80 mg/mL extract. The quick recovery rate of animals was observed at least concentrations, while the rate of recovery was found to be delayed at higher concentrations. Nevertheless, the animals reverted back to normal state at all the concentrations of extract tested. Percentage of recovery is provided in Fig. 1B. Rapid onset of anesthetic effect at lower concentration of plant extract *Ottonia Propinqua* (Piperaceae) was reported by Rates et al. (1997). Similarly, intradermal wheal method experiment performed in guinea pig and humans by Alawdi et al. (2019) reported that 100 and 50 mg/mL of *Chrysanthemum cinerarifolium* extract has the ability to elicit local anesthesia effect without any adverse outcome. The authors also compared the results with the lidocaine (a local anesthetic agent) prepared from the plant extract found equivalent results with *C. cinerarifolium* extract. In another study from Muralikrishnan et al., (Muralikrishnan et al. 2017) the efficacy of *Anacyclus pyrethrum* root extract was evaluated for its local anesthesia activity in guinea pigs. The animals injected intradermally with plant extract revealed significant local anesthetic effect at 2% ethanolic root extract of *A. pyrethrum* with rapid onset of time. Similarly, an intradermal injection of extract of of *Sterculia tragacantha* Lindl displayed local anesthetic activity in guinea pig (Udegbunam et al. 2012). Plant extract consists of numerous bioactive molecules, such as alkaloids, saponins, and terpenoids, and therefore, it is not surprising that the extract of *O. anisum* exhibiting local anesthesia activity in guinea pigs.

### Effect of O. anisum root extract on ALI

#### Effect of O. anisum root extract in HCl-induced ALI mice

*O. anisum*, a medicinal plant with numerous phytoconstituents, has been used in the fold medicine to treat several illnesses. In the present research the root extract was assessed for anti‑inflammatory potential using mice model of HCl‑induced ALI. Mice were administered orally with different doses of *O. anisum* root extract and the response to each concentration were studied in ALI mice. The extract was administered 90 min before HCl-instillation. The lung tissue homogenate from positive control (HCl-instilled mice), negative control (no HCl and extract administration), and *O. anisum* extract treated mice were assessed for BALF, inflammatory cells, neutrophils, redox homeostasis, ROS generation, lipid-peroxidation marker MDA, catalase, reduced GSH and quantitative gene expression of pro‑inflammatory genes.

**Fig. 2:**
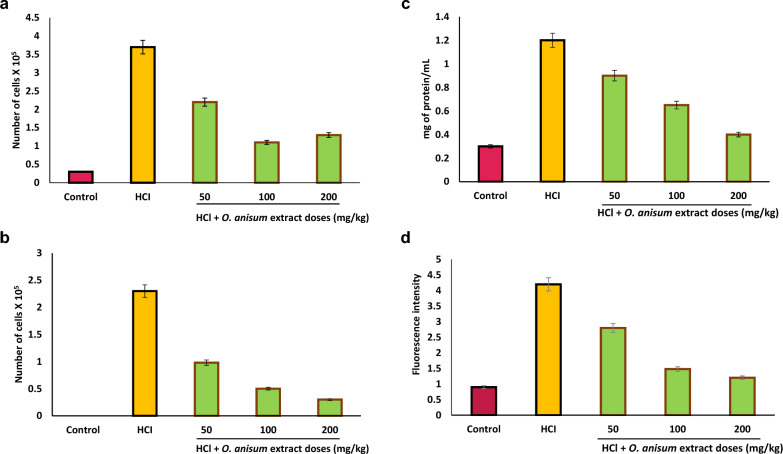

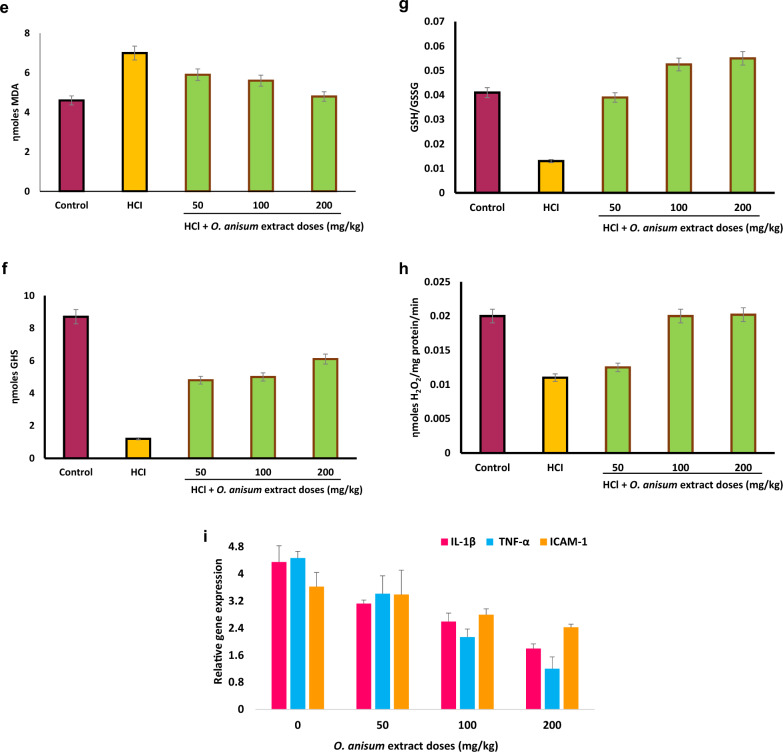
Effect of *O. anisum* root extract in HCl-induced ALI mice. Different doses ranging from 50 to 200 mg/kg were assessed in ALI mice. Animals subjected to HCl served as positive control, while animals received only food and water served as negative control. **A** Total number of cells from BALF of animals treated with and without *O. anisum* extract. **B** Total number of neutrophils from BALF in the presence and absence of *O. anisum* extract. **C** Total protein content from BALF of animals treated with and without *O. anisum* extract. **D** ROS level in the presence and absence of the *O. anisum*. **E** MDA levels of animals treated with and without *O. anisum* extract. **F** The reduced GSH levels of animals treated with and without the extracts in HCl-induced ALI mice. **G** The levels of redox homeostasis in the presence and absence of *O. anisum* extract. **H** Catalase activity in the presence and absence of *O. anisum* extract. **I** *O. anisum* extract down regulates the expression of NFkB-associated genes in the lungs of HCl-induced mice. The lung tissue was collected from the positive control (HCl exposed) mice and *O. anisum* extract treated mice. Total RNA was isolated and purified from the control and treated lung samples and converted into cDNA. The cDNA was assessed for the pro-inflammatory associated gene expression (IL-1β, TNF-α, and ICAM-1) using their respective primers. β-actin, a reference gene, used to normalize the gene expression. Data are denoted as mean ± SD (*n* = 6)

Group III mice administered orally with *O. anisum* extract before HCl treatment was found to have reduced number of inflammatory cells in BALF. An increase in the total cell count signifies the ALI conditions. Figure 2A represents the total number of cells from BALF in the presence (50 mg/kg, 100 mg/kg, and 200 mg/kg) and absence of the *O. anisum* extract. As a hallmark of ALI, inflammatory cells in the BALF were raised to 3.7 × 10^5^ cells (*P* < 0.001) in the case of HCl treatment. However, mice administered with different doses of extract before HCl treatment showed considerable reduction in the number of inflammatory cells. Among the doses tested, a dose of 50 mg/kg, i.e., the minimal dose of the extract was noticed sufficient to restore the normal number of inflammatory cells. A saturation point was observed at 100 mg/kg *O. ansium* extract and there is no considerable increase of cell count was observed after 100 mg/kg. These results denote the anti-inflammatory potential of *O. anisum* against HCl-induced ALI mice (Kaur et al. 2018).

Figure 2B represents the total number of neutrophils from BALF in the presence and absence of *O. anisum* extract. HCl administration in mice has caused a sudden increase in neutrophil counts in the BALF. Interestingly, the mice treated with 50 mg/kg dosage of *O. anisum* extract before HCl administration showed significant reduction (*P* < 0.001) in the neutrophil count, clearly depicting the positive effect of *O. anisum* extract. Further increase in concentration of *O. anisum* extract (100 and 200 mg/kg), completely suppressed the neutrophil count. From Fig. 2b, it is clear that 100 mg/kg dosage is sufficient to reduce the neutrophil counts hugely in the lungs (Kaur et al. 2018).

Figure 2C represents the total protein content from BALF of animals treated with and without *O. anisum* extract. HCl administered mice were found to have increased protein content in comparison with control group animals. However, treatment with *O. anisum* extract before HCl administration, reduced the protein content in the BALF. From Fig. 2C, the reduction in protein content was found to be comparatively lower at maximal concentration (*P* < 0.001), than minimal concentrations of *O. anisum* extract. These results suggest that 200 mg dosage could reduce the pulmonary edema to a significant level (Kaur et al. 2018).

#### Examination of O. anisum extract on oxidative stress of ALI mice

*O. anisum* extract was found to effectively regulate the ROS and lipid peroxidation levels in HCl administered mice.

##### Effect of O. anisum extract on ROS

Figure 2D represents the ROS level in the presence and absence of the *O. anisum.* Mice administered with HCl showed significant increase in the ROS levels (*P* < 0.001) in comparison with control mice group. The escalation in ROS level indicates the oxidative stress generated by HCl administration. Mice treated with *O. anisum* extracts before HCl administration greatly reduced the ROS amounts (*P* < 0.001) in the lungs. However, maximal reduction of ROS levels was observed at 200 mg/kg dosage (Kaur et al. 2018).

##### Effect of O. anisum extract on MDA

MDA serves as the potential biomarker for lipid peroxidation. Measurement of MDA directly signifies the cell injury due to oxidative stress and free radicals. Therefore, mice treated with *O. anisum* extracts were analyzed for MDA levels. Figure 2E represents the MDA levels of animals treated with and without *O. anisum* extract*.* Here, mice administered with HCl showed increased levels of MDA in assessment with control mice (*P* < 0.001). However, animals treated with different concentrations of *O. anisum* extract showed decrease in MDA levels (*P* < 0.01) in correlation with mice administered with HCl alone (Kaur et al. 2018). As evident from Fig.  2E, 100 mg/kg dosage was found to be the most effectual dose, as the levels of MDA was significantly reduced at this concentration (*P* < 0.001).

#### Evaluation of O. anisum extracts on redox homeostasis and catalase activity of ALI mice

Redox homeostasis is defined as the equilibrium state between oxidations and antioxidations and catalase is an important antioxidant enzyme that protects the cells from ROS destruction during oxidative stress. Therefore, effect of *O. anisum* extract on redox homeostasis and catalase activity were investigated in the presence and absence of extracts. Results unveiled that mice treated with *O. anisum* extract was found to restore the redox homeostasis and catalase activity in lungs (Kaur et al. 2018).

GSH, an antioxidant that prevent cellular damage, was found to increase in the presence of *O. anisum* extract. Figure 2F represents the reduced GSH levels of animals treated with and without *O. anisum* extracts in HCl-induced ALI mice. A significant reduction in reduced GSH levels (*P* < 0.001) was observed when compared to the levels of reduced GSH in control mice. Intriguingly, the mice treated orally with different ranges of concentration of *O. anisum* extract unveiled the dose-dependent activity in reduced GSH levels. These results suggest that *O. anisum* extract could significantly revert the GSH levels in HCl-mediated ALI mice.

The ratio of oxidized/reduced GSH was used to analyze the redox homeostasis in lungs. Figure 2G depicts the levels of redox homeostasis in the presence and absence of *O. anisum* extract in HCl-induced ALI mice. A dose-dependent increase in redox homeostasis was observed in animals remedied with different concentrations of *O. anisum* extract when compared to mice administered with HCl alone. This result clearly depicts the positive effect of *O. anisum* extract on redox homeostasis in HCl-induced ALI mice.

Figure 2H represents the catalase activity in the presence and absence of *O. anisum* extract in HCl-induced ALI mice. When compared to control group, mice administered with HCl showed decreased catalase activity (*P* < 0.001), indicating the oxidative stress and low level of antioxidant enzymes in HCl administered mice. However, in the presence of extracts the catalase activity was significantly increased. The catalase activity was restored to normal at 100 mg/kg and 200 mg/kg dosage (*P* < 0.001).

#### RT-PCR analysis of pro-inflammatory associated genes

RT-PCR results unveiled the down regulation of essential genes associated with ALI in the presence of *O. anisum* extract. Figure 2I represents the gene expression of pro-inflammatory associated genes (IL-1β, TNF-α, and ICAM-1), which is highly regulated by NFkB proinflammatory factor. IL-1β gene expression is comparatively higher in HCl administered mice in comparison with control mice group. Animals administered with 100 mg/kg dosage of *O. anisum* extract before HCl treatment, showed down regulation of IL-1β and TNF-α, in comparison with HCl instilled mice group. However, there is no major difference exist between ICAM-1 gene expression in group II and group III mice (Kaur et al. 2018). Suppression of key genes associated with ALI indicate the significant role of *O. anisum* extract in lung diseases.

### Analgesic activity of O. anisum extract

**Fig. 3:**
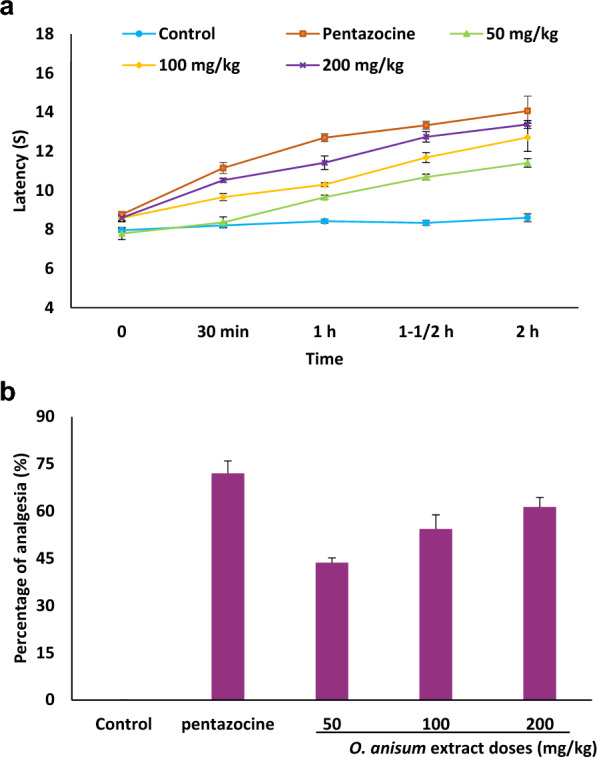
Evaluation of analgesic activity of *O. anisum* extract in rats. Different doses ranging from 50 to 200 mg/kg were assessed in rats. Animals injected intraperitoneally with pentazocine served as positive control. **A** Latency time in seconds after intraperitoneal administrations with *O. anisum* extracts. **B** Percentage of analgesia activity at different dose of *O. anisum* extract. Data are denoted as mean ± SD (*n* = 6)

Analgesic activity at different doses of *O. anisum* extract (50–200 mg/kg) was determined through Eddy’s hot plate method. The analysis revealed the increased latency time with significant analgesic activities (Fig. 3A, B). The rats injected with pentazocine exhibited considerable analgesic activity (72%). The study finds dose-dependent upliftment of latency time with maximal analgesic activity at 200 mg/kg of *O. anisum* extract. Comparable results were described from Ghauri et al. (2021) in which the methanolic plant extract of *Euphorbia granulata* revealed substantial analgesic activity at 200 mg/kg. Relatedly, Reza et al. (2021) have reported maximal activity at 200 mg/kg from *Aeginetia indica* methanol extract. Malairajan et al. (2006) have reported analgesic activities from various Indian medicinal plants.

### Evaluation of anti-inflammatory activity

**Fig. 4:**
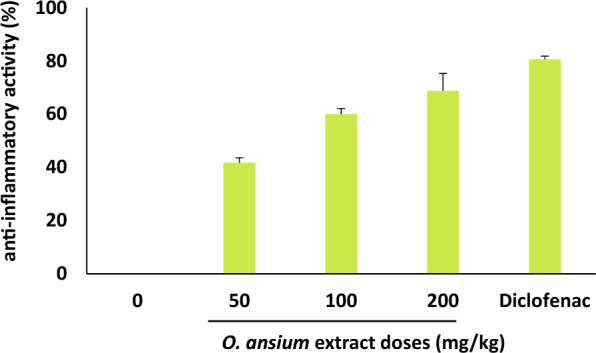
Evaluation of anti-inflammatory potential of *O. anisum* extract in rats. Different doses ranging from 50 to 200 mg/kg were assessed in rats. Animals treated with dichlofenac served as positive control. Data are denoted as mean ± SD (*n* = 6)

Analysis of *O. anisum* extract on anti-inflammatory activity through carrageenan paw oedema method revealed the inhibitory activities in extract treated rats and diclofenac treated rats. Dose-dependent inhibitory activity was observed and the maximal inhibitory activity was monitored at 200 mg/kg of *O. ansium* extract. On the other hand, the significant inhibitory activity was observed in diclofenac treated rats (Fig. 4). However, the anti-inflammatory activity of methanolic extracts of various plants such as *Mollugo cerviana, Citrus nobilis, Palicourea crocea, Callicarpa arborea Roxb* have been demonstrated previously (Antony et al. 2022; Malik et al. 2021; Formagio et al. 2019; Ema et al. 2023). Furthermore, The exhibited anti-inflammatory properties may be attributed to the presence of bio active compounds in plant extract. In addition, the bioactive components within the extract may play a role in stabilizing membranes by potentially suppressing the release of lysosomal constituents from neutrophils at sites of inflammation (Malik et al. 2021).

## Conclusions

The present study reports the local anesthetic, analgesic and anti-inflammatory effect of *O. anisum* extract as well as the therapeutic efficacy of *O. anisum* extract in HCl-induced ALI mice. Local anesthetic activity in the presence of different concentrations of *O. anisum* extract revealed the dose-dependent anesthetic effect. Furthermore, the ALI studies unveiled that the extract could curb the activation of NF-KB, a key factor responsible for developing ALI as well as the ability to restore the redox homeostasis in HCl-induced ALI mice. However, further research in human ALI is anticipated to reveal the complete therapeutic efficacy of *O. anisum* extract. Furthermore, appraisal of analgesic and anti-inflammatory capability of *O. anisum* extract unveiled the significant activities in rats. Therefore, this study revealed the future prospective of *O. anisum* extract towards biomedical applications.

## Data Availability

Data are available upon request from corresponding author.
